# Conveying the need for mental healthcare – a qualitative study of how patients communicate mental health challenges

**DOI:** 10.1186/s12913-025-12851-1

**Published:** 2025-05-10

**Authors:** Marit Nymoen, Marit Synnøve Lomeland, Eva Biringer, Mirjam Ekstedt, Øystein Hetlevik, Rod Sheaff, Olav Thorsen, Miriam Hartveit

**Affiliations:** 1 Department of Research and Innovation, Fonna Hospital Trust, Haugesund, Norway; 2 Department of Global Public Health and Primary Care, Faculty of Medicine, University of Bergen, Bergen, Norway; 3 The Bipolar Association, Tysvær, Norway; 4 Department of Health and Caring Sciences, Faculty of Health and Life Sciences, Linnaeus University, Kalmar, Sweden; 5 Peninsula Medical School, Faculty of Health, University of Plymouth, Plymouth, UK; 6 The General Practice and Care Coordination Research Group, Stavanger University Hospital, Stavanger, Norway

**Keywords:** Communication, Referral and consultation, Secondary care, General practitioner, Specialised mental healthcare

## Abstract

**Background:**

Access to timely mental healthcare relies on patients’ descriptions of their mental health problems. We therefore sought to better understand, from the patients’ perspective, how they communicate their need for specialised mental healthcare to their GPs or mental health specialists and what factors affect communication when patients are referred from their GPs to specialised mental healthcare.

**Methods:**

This was an exploratory interview study. Ten adults who started treatment in specialised mental healthcare facilities were interviewed individually. The interviews were audiotaped and transcribed verbatim. A method based on thematic analysis was used to develop patterns and themes within the dataset using an iterative inductive approach, with checks for internal consistency throughout.

**Results:**

Three typical personal approaches – or styles – of communicating needs could be generated. These approaches varied in how active the patients were in their help-seeking, how unrestrictedly they communicated their health concerns and their receptiveness to input from healthcare professionals. Relevant factors affecting the communication were the characteristics of the healthcare services; the responses of others; fear of rejection and misunderstanding; health literacy and experience with mental healthcare; taking responsibility for one’s own treatment; and the mental health problem itself.

**Conclusions:**

The different patient approaches to getting help for mental health problems and how those approaches are affected by individual, contextual and system factors highlight the need for individualised and welcoming communication by care providers. The current study contributes with useful insights from the patient’s perspective into how e.g. the patient’s previous experiences and understanding of the healthcare system influences the process of seeking help from a GP and being referred to specialist mental health services.

**Supplementary Information:**

The online version contains supplementary material available at 10.1186/s12913-025-12851-1.

## Introduction

Tailored and timely specialised mental healthcare depends on the quality of communication between the patient and their GP concerning the patient’s healthcare needs [1–3] because the GP must obtain appropriate and sufficient information to make reliable and valid assessments of the need for such services [4–7]. In Norway, as in other countries with a two-level health care system, the process of referral from the GP to a mental health specialist usually begins with communication between the GP and patient before the GP writes a referral letter [8–10]. Based on the information provided in that letter, specialists decide whether the patient should receive treatment, what treatment they should receive and the maximum acceptable waiting time given the patient’s symptomatology and situation [8, 11, 12].

What information is considered important to convey during the referral process depends on the perspective of each stakeholder because GPs, patients and specialists define patients’ needs for specialised mental healthcare somewhat differently [2]. A previous study reported that, for half of patients, specialists’ assessments of the timeliness of care were different when based on referral letter information than on a patient consultation [13]. These results [13] suggested that there is a risk of a patient receiving care that would be considered untimely by health professionals or patients, which has been linked with worsening physical health and an increased risk of poor prognosis, self-harm and suicide [14–17]. Unclear or insufficient communication also creates the risk of a help-seeking person being rejected by mental health specialist services or of treatment interventions being provided that are not tailored to their needs [3, 4, 18].

A patient’s recovery process can be facilitated by a mutual understanding of their needs and by appropriate communication and collaboration between the patient and healthcare professionals [19], while patient well-being, patient satisfaction and health status can be directly influenced by improvements in GPs’ verbal and nonverbal communication in consultations [20–22]. However, even talking about emotional problems when seeking help from a GP can be experienced as difficult by the patient [23], and confusion about the causes of the patient’s healthcare situation can represent another barrier to communication [23]. Conversely, a ‘human connection’, expressed as empathic behaviour by the GP, and efforts to reach a shared understanding between the patient and GP have been found to facilitate communication [23].

In the current study, we sought to explore, from the patients’ perspective, how they communicate their needs for specialised mental healthcare to their GPs or mental health specialists and what factors affect communication of such needs when they are referred to specialised mental healthcare.

## Methods

### Context

In Norway, public mental healthcare is provided in both primary and specialised care settings [24]. Primary healthcare often consists of the patient’s GP and low-threshold mental healthcare services in municipalities [25], while specialised care is provided by second-tier community mental healthcare centres (CMHCs; in Norwegian, Distriktpsykiatriske sentre [DPS]) and third-tier psychiatric hospitals [24, 25], which are responsible for patients from several municipalities.

GPs, psychologists in primary healthcare, private healthcare doctors and certain other service providers can refer patients to mental health specialist care, most often by a written referral letter. The triage of patients referred to mental health specialist care is performed by hospital or CMHC specialists [8]. In Norway, prioritisation guidelines assist hospital specialists’ assessments of patient triage and provide suggestions for maximum acceptable waiting times before treatment starts for patients with different health situations [12]. How effective the treatment is and the cost-effectiveness of the intervention are the two main criteria for determining whether a patient should receive specialised mental healthcare [11].

Most often, the triage is based solely on information provided in the written referral letter from the GP [8]. Approximately 20% of all referrals to specialised mental healthcare in Norway are rejected [26], and it is therefore important that GPs’ descriptions of patients’ healthcare needs and situations are correct and in line with the perceptions of both the patients and GPs. Critically, GPs mostly rely on information from and the perceptions of the patient when writing a referral letter.

### Design

An exploratory design with individual patient interviews was chosen to address the study’s aims because patients’ experiences and perceptions of communicating their needs with their GPs are deeply personal and subjective; patients are thus more likely to disclose them in individual rather than group interviews. The patients were interviewed after starting specialised mental healthcare treatment to allow data to be gathered on how they experienced communication of their needs both during GP consultations before referral and at the CMHCs after the start of specialist treatment.

### Recruitment and sample

All patients attending their first appointment at either of the two collaborating CMHCs were invited to participate. The inclusion criteria were being 18 years or older, receiving outpatient specialised mental healthcare and having been referred by their GP or the emergency department of a general hospital directly to a CMHC.

Patients wanting to participate contacted the first author by e-mail, letter or phone/text message to express interest. The researchers then contacted them to provide further details about the study, secure consent and determine a time and place for the interview. This method of recruitment therefore represented an inclusion criterion by including only patients who actively wanted to share their experiences.

We interviewed three men and seven women. Eight of the ten interviews were held at the CMHCs, with the remaining two conducted by video conference, one because of distance from the CHMC and the other for practical reasons. The participants were invited and the interviews conducted between June and November 2023. Given the nature of the individual experiences and the diversity of the patients’ situations, we did not seek data saturation [27]; instead, we sought to gather rich data from a small sample in line with the recommendations of Malterud et al. [27]. Prior to the study, we expected 8–10 patients to be sufficient.

Approximately 345 patients received an invitation to participate. At one of the CMHCs, medical secretaries were responsible for providing written information about study participation, while at the other, healthcare personnel meeting patients during their first consultations provided information about study participation. Posters with information about the study and contact information were also placed in the waiting areas of both CMHCs.

Two researchers interviewed each person. To increase confidentiality, all participants are described as they/them. Four participants were between 18 and 30 years old, three between 31 and 50 years and three between 50 and 65. All the participants had previously received some form of mental healthcare; some had only received diagnostic examinations or easily accessible primary mental healthcare, while others had mainly received help in specialised care or from private mental health specialists. There were five participants from each CMHC.

### Data collection

To facilitate the interviews, a semi-structured interview guide was developed for this study with open-ended questions such as ‘What was important for you to communicate to your GP when you sought help?’ and ‘What has been helpful or a hindrance in describing your health issues in a clear way?’ (see the appendix for the full interview guide). The three interviewers (MN, MSL, MH) asked follow-up questions as appropriate to develop a deeper understanding of the topic.

The group conducting the interviews comprised one author with lived experience as a service user, one who was a healthcare services researcher and one who was a clinical psychologist. All three participated in developing the interview guide, which helped them to maintain the same focus in all interviews, as only two interviewed each participant: either the researcher or the clinician moderating the interview and the service user as an observer. The semi-structured interviews lasted approximately 45–60 min and, with the patients’ consent, were audiotaped and transcribed verbatim.

### Data analysis

We used a method based on thematic analysis [28], which is a data-driven approach, to generate themes in the data in an iterative, inductive process. Three of the authors (MSL, MH, MN) first read the transcripts and wrote summaries of each transcript (phase 1 in the outline of thematic analysis by Braun and Clarke [28]). These summaries were a foundation for reflexive dialogues in which the three researchers discussed various interpretations of the data. From each discussion, the first author summarised the main underlying meanings or interpretations of the data in preliminary themes and subthemes. These early discussions were introduced to increase the possibility of important information not being lost and all three perspectives (i.e. service user, healthcare professional and healthcare services researcher) being included early in the analysis process. Further, the first author reviewed the transcripts and the summaries from the group discussions, and made suggestions for categories and themes, in line with phase 2 and 3 described by Braun and Clarke [28]. This iterative process combining group discussions and creation of themes was continued until reaching a consensus concerning the constructs underlying the interviews and agreement on the final themes and subthemes (phase 4 [28]). All authors took part in the last stages of the discussion concerning how the results and constructs could be understood (phase 5 and 6 [28]).

Seven preliminary main themes were developed, which were refined in the group discussions after reading all the summaries. Final agreement was reached on six main themes for the patients’ experiences of communication and three for characteristic communication styles. Involving multiple researchers in the analysis helped to address dependability because individual interpretative repertoires can vary [29] and alternative interpretations can be reached.

### Ethics

The Norwegian Agency for Shared Services in Education and Research (Sikt) approved the study as consistent with the existing legal and ethical frameworks (ref. no. 612290). All patients volunteered to be interviewed and were informed that their treatment in specialised mental healthcare would not be influenced by their choice of whether to participate. Personal data were removed during transcription. The study was fully financed by the Fonna Hospital Trust, Norway, via the salaries of the authors.

## Results

Ten unique experiences and perceptions of seeking help for mental health problems and communicating needs were described in the individual interviews. Based on the patients’ descriptions, we derived three types of personal approaches – or personal styles – related to how open-minded and active the patients were when asking for help. These approaches were affected by six contextual factors: the characteristics of the healthcare services; the responses of others; fear of rejection and misunderstanding; health literacy and experience with mental healthcare; taking responsibility for one’s own treatment; and the mental health problem itself. According to the participants, their approaches to communication were dynamic; that is, their approaches changed during the help-seeking process in response to new insights, setting-related factors and healthcare professionals’ responses to their needs.

In the first style, the participants described their problems unrestrictedly; they appeared open-minded to suggestions from healthcare professionals about what treatment may be most suitable, but they often depended on support from others in seeking healthcare. We termed this style of communication *open-minded and passive*. Typical of this approach were descriptions of both how others helped the participants realise that they could benefit from healthcare services and uncertainty concerning their own needs, usually alongside acceptance of the care they were offered. This approach to communicating mental health challenges might be changed by, for example, the participant becoming more knowledgeable about their own mental health. One participant described how they were gradually able to better understand and describe their challenges to their GP and how they actively sought help only after some time, asking the GP directly for more help.

In the second style, the participants described taking a more active approach in asking for mental healthcare. According to them, they talked openly about their problems and were open to expert opinions from healthcare professionals. We termed this approach *open-minded and active*. Some had talked with friends or family about their challenges and then refined how they described their understanding of their own healthcare needs as their insights into those needs deepened:

*Talking [to friends/family]*,* in a way*,* about [the mental health challenges] a lot means that you catch all the little details*,* and then you can catch the nuances*,* distinguish things*,* see slightly clearer lines between it all*,* put more words to the specific things that may be a problem.* (Participant 3)

However, this approach could be affected by how the GP responded. One participant described being initially open in their communication with the GP, but when their mental health challenges were downplayed by the GP, they stopped contacting the GP and tried to handle the challenges alone, such as by reading about the suspected diagnosis.

In the third style, the participants described an approach that we termed *determined and active*. Typical of this approach was a clear understanding of the kind of help needed, and, therefore, the information provided to the GP or specialist was restricted to what the participant believed was relevant for the healthcare professional to know to provide the necessary care or referral. These patients were also less open to care providers’ opinions and suggestions for alternative interventions. Their aim was to persuade the healthcare professionals of their need for treatment; they described asking for a service, such as a diagnostic examination or treatment for a specific diagnosis, and some were very forward in demanding help for their mental health problems:

*So*,* fortunately*,* I recognised the danger signs*,* and I put my foot down and absolutely screamed: ‘You have to refer me now!’ If I’d heard … I’m just thinking … if I’d been 17 or 18 and had no idea what you’re entitled to*,* I’d have really struggled.* (Participant 2)

Typical of the participants taking this approach was knowledge of mental health issues, either from previous treatment or by acquiring knowledge about illnesses on their own. One participant, however, described how diagnostic examinations at the CMHC led to new ways of understanding their struggles. Their assumptions were mildly challenged by their having to complete different inventories, and they became more open to different ways of interpreting their symptoms.

### Barriers and facilitators to the effective communication of mental health challenges

Based on the transcripts, we developed six themes to describe the participants’ perceptions of what affected the communication of their experienced need for mental healthcare when seeking help from a GP or mental health specialist. These themes were *characteristics of healthcare services; responses of others; fear of rejection and misunderstanding; health literacy and experience with mental healthcare; taking responsibility for one’s own treatment;* and *the mental health problem itself.*

#### Characteristics of healthcare services

Several characteristics of healthcare services, especially how participants understood how healthcare services ‘work’, affected the participants’ experience of communicating their mental healthcare needs to their GPs. Perceptions about what information to share and with whom were connected to how the GP’s role was understood by the patient and the length of their consultations at the GP’s office. Limited time meant that the participants had to prepare themselves for the consultation by, for example, writing down what they needed help with beforehand. Some participants also did not expect the GP to be a mental healthcare provider but rather a stepping stone towards specialised mental healthcare. A few of those participants who held this view stated that they adapted the information they provided when meeting the GP to obtain a referral to specialist services as easily as possible. They sometimes even left out information about their main healthcare needs because they did not see why the GP should have that information. One participant said they provided less information to the GP to protect themselves from sharing emotionally ‘heavy’ details that they felt should be shared later with the specialist at the CMHC because they did not expect the GP to provide mental healthcare:

*I didn’t want to go into depth with [the GP] about everything. *(Participant 3)

This and other participants described how they explained everything to the specialist without modifying the information because they regarded a secondary care facility as the correct place to receive mental healthcare.

A couple of participants mentioned how their own or the GP’s adaptation of the information led to information in the referral letter, such as the patient having a diagnosis, that was ‘wrong’ and that could affect the treatment offered in secondary care. One participant stated a clear concern about the GP’s adaptation of information, in which the GP added details that the participant did not agree with:

*And [the GP] said that to me beforehand*,* just like*,* ‘Yeah*,* I’m also writing something like emotionally unstable personality disorder [in the referral]*,* because if I write a lot of things*,*’ she said*,* ‘there is a greater chance of getting in [to the CMHC].’* (Participant 10)

#### Responses of others

The participants said that the tone for later meetings was set by how they were met at the first meeting – for example, at the GP’s office – regarding their mental health needs. Experiences of not being taken seriously, GP behaviour that was described as negative and not being asked relevant questions or indeed any questions at all could result in the participant not daring to explain the situation further or even to mention mental health challenges in the first place, thus preventing communication of the need for healthcare. The participants who experienced not being taken seriously did not ask for more help with their mental health challenges but rather tried to handle the challenges by themselves, waited for the right GP substitute or registered for a new GP.

Participants described how not being taken seriously made them feel that they were not being listened to regarding the seriousness of their symptoms. Some sought help from other healthcare services, both privately financed care and alternative primary healthcare services, such as more easily accessible support from psychiatric nurses. These participants actively sought help, while others were more passive in their help-seeking. Some participants described waiting years until they felt it was the ‘right’ time to tell their GP about their problems and ask for services. Meeting a new GP or substitute who took them seriously was a trigger for one participant to talk about their mental health issues:

*I mean*,* right there and then*,* it was very spontaneous*,* actually very kind of emotional … I feel safe here to talk and discuss it [with the GP substitute]. … Now it’s time for me to admit that I have a problem that I can’t manage to fix properly myself. (Participant 6)*

Other participants stated that a GP taking active, tangible steps to help them, such as by ordering a blood test in connection with a referral to specialised mental healthcare, made them feel understood, which made it easier to communicate their needs to the GP. Being invited to take part in central decisions about their personal care pathway also strengthened the feeling of being taken seriously by the GP and hospital specialists. One of the participants provided the following example of how a GP responding to their request for help with mental health challenges strengthened their sense of being taken seriously:

*[The GP] was very*,* like*,* right away*,* ‘What specifically can I do*,* and what are the next steps to get this up and running?’ And I think that*,* I mean*,* for me*,* that was brilliant*,* because then it was very*,* like … and I said*,* ‘I fully understand that we are talking here about a problem that will take time*,* but I want to get started*,*’ and she really agreed.* (Participant 6)

#### Fear of rejection and misunderstanding

Fear of the referral being rejected or of the patient being discharged by the hospital specialist earlier than the patient wanted could, in some cases, shut communication down and, in others, encourage them to share more of their personal story. One participant mentioned that they did not dare to start explaining how they felt if they did not believe that the GP would take them seriously. Another reported that they had been rejected when referred to specialised mental healthcare earlier because they looked ‘too well’ physically and were later afraid that they might not be taken seriously if they sought help again. Other participants received information about a possible rejection at the CMHC, which encouraged them to be very open with the hospital specialist about their challenges. For example, one participant became determined to communicate their mental health issues in a clearer way to the specialist when they received information that they were being assessed for specialised mental healthcare and could be rejected:

*I’ve come up with a sort of very simple explanation that maybe can help [the hospital specialist] a bit to see what the problem is*,* so … to try to relate a bit to something that he actually might recognise in himself*,* that’s probably the easiest way to get it across*,* I think. (Participant 8)*

Some participants reported experiences of being misunderstood or not understood at all by healthcare professionals. Self-report inventories and structured interviews were seen as useful for some participants for avoiding this type of misunderstanding. One participant however stated that they did not trust the self-report inventories that were used for diagnosis and therefore did not answer them:

*So*,* when I was in [inpatient care]*,* they gave me a form with 500 questions on … I can honestly say that they just had to take it away. … If you put ‘yes’*,* then [the healthcare personnel] start creating their own opinions and viewpoints*,* and I think that’s just stupid.* (Participant 5)

#### Health literacy and experience with mental healthcare

The participants reported that knowing more about healthcare and health literacy often led to an unrestricted communication style with both GPs and specialists. For example, one participant mentioned that having identified the best treatment option and most relevant CMHC for their healthcare needs made it easier to ask for help:

*For me*,* it was when I investigated and saw that there were [mental health treatment centres] in lots of places*,* including in [first town] and [second town]; that was actually a point that made it quite an important discovery for me because then I had something specific to ask about – being referred there.* (Participant 6)

One participant described how they had previously received several types of treatment but none had fit their needs. These experiences of ineffective treatment made them ask for something other than the ‘standard and recommended treatment’, and they described communicating this to the hospital specialist:

*So*,* when I come to the therapist*, *I always explain who I am*,* but even so*,* it’s cognitive therapy [they offer me]*,* here and now. But I’ve said to them*,* ‘We have to go back and see who I am*,*’ but ‘no*,* no*,* no*,*’ you always have to start from here and now.* (Participant 9)

One participant described how their experiences when working in mental healthcare made them more prepared to read and understand their own patient records and understand what, for example, the hospital specialist would like to know more about when beginning treatment in secondary care.

#### Taking responsibility for one’s own treatment

According to the participants, taking responsibility and ensuring that health professionals received what the patients considered correct information could improve clarity of communication with both the GP and hospital specialists. It could also lead to the communication being adapted to the desire for treatment and to expectations about how the professionals would react, as described above. One way that taking responsibility impacted communication was when the patients read their own records online to keep up with what the hospital specialist wrote and thus ensured that the information that the specialist received from the GP and patient was understood and correctly recorded. Fears of negative consequences for later treatment or of ‘wrong’ diagnoses – from the patient’s perspective – were often the reasons for checking records. One participant took responsibility by making a request to have the information in their record changed because they believed it was erroneous.

*The one psychologist even wrote all sorts of stuff in the patient record that’s not right at all*,* and there are quite a few record entries in there*,* and I’ve made a complaint. (Participant 5)*

#### The mental health problem itself

The way participants conveyed their challenges was sometimes affected by the symptoms of their mental health problem. Being in a state of crisis or experiencing chaos could, for example, make a participant more ‘childish’ in expressing themselves:

*Just when the feelings set in*,* it’s just like being ten years old again*,* and when you look at it afterwards*,* you feel even more ashamed*,* and then it’s just that you want to suppress it even more*. (Participant 1)

One participant also revealed that, when they were at their lowest point, they were assessed in a consultation at the CMHC to determine whether they should receive secondary healthcare. They experienced this consultation as an ‘audition’ in which they met three hospital specialists who, after some time, discussed between themselves what type of treatment the participant should receive. The participant described the experience as negative – having to ‘perform’ and be triaged then and there – and how they struggled to describe their situation appropriately because of their mental health challenges.

## Discussion

The aim of this study was to explore how patients communicate their need for mental healthcare to their GPs and mental health specialists and the factors that may affect that communication from the patient’s perspective. Three typical personal approaches related to activeness of help-seeking, restrictedness of communication of health needs and receptiveness to input from healthcare professionals were generated, and six themes regarding what could affect patients’ communication of their mental healthcare needs were developed: *characteristics of the healthcare services; responses of others; fear of rejection and misunderstanding; health literacy and experience with mental healthcare; taking responsibility for one’s own treatment;* and *the mental health problem itself*.

### Implications and comparison with existing literature

The results show that some patients cannot or are hindered from communicating their healthcare needs appropriately and comprehensively because of, for example, the characteristics of the healthcare system, negative experiences with health professionals and how their mental health challenges affect their ability to communicate. These findings suggest that communication between the patient and GP influences later triage at the CMHC because the GP may not receive the information the patient regards as important and thus cannot include it in the referral letter. This may explain the results of a recent study that found that the quality of referral letters was not associated with the reliability of waiting time assessments [13].

Participants in the present study described how they and sometimes the GP adapted information, either in verbal communication or in the referral letter, to obtain the desired treatment from the mental health specialist or to avoid having the referral rejected. This was also found in a previous study [2] in which patients adapted information when consulting their GPs to either increase or decrease the chances of receiving specific healthcare. GPs must therefore be aware of such adaptations and seek to identify what information might have been modified.

The current study suggests that there are several ways communication between patients and healthcare professionals may be facilitated or hindered. For example, professionals should keep in mind that previous experiences with themselves or with other healthcare professionals may have been negative and that the threshold for patients to even describe their mental health issues can be high. The participants also described not being taken seriously, which highlights the need for empathic, active listening when a patient describes their needs – a finding supported by other studies [3, 23, 30].

Healthcare professionals should also seek to facilitate patient communication [21, 23, 31, 32] by e.g. aiming for shared understanding with the patient and provide recommendations based on this understanding [21]. As the results of this study shows, efforts to facilitate the communication can affect how much information patients provide. The need for professionals to facilitate communication is further highlighted by a recent study that found that approximately a quarter of patients received longer maximum waiting times before treatment when the assessment was based on a referral letter than when a specialist made an urgency assessment after the first patient consultation [13].

Different approaches to communicating the need for mental healthcare also affect how healthcare professionals can facilitate communication. The present study found that patients with passive attitudes may not take the initiative to communicate their needs and may need more facilitation than those who are actively help-seeking with an unrestricted communication style. However, patients who are determined about their need for treatment and unreceptive to suggestions from healthcare professionals can ‘challenge’ the referral system. Further studies exploring how patients with this style of communication can be helped by healthcare professionals to receive the most appropriate care are needed. For example, one article underlined how tailoring care to the patient and being sensitive to how one communicates is key to improve quality in health services [33], and might be relevant for patients who are determined about their need for treatment. Further studies should also be conducted to address the limitations of the sample in this study, including its size, demographic range, and sampling method, as described below.

### Strengths and limitations

None of the patients were over 65 years of age, and the sampling method inherently excluded those who were rejected when referred to specialised mental healthcare. The sample was also rather small, and, therefore, although we generated three characteristics for understanding the need for mental healthcare, there may be several combinations and other characteristics that we did not generate. The recruitment method also excluded participants who did not take the initiative to participate, and we therefore lack information on these patients’ experiences.

The study used an analysis process based on thematic analysis [28, 34], with a particular emphasis on the iterative process in which the codes, categories and themes were continuously compared with transcripts to increase the internal validity of the study, which may have increased the confirmability of the results [35].

The researchers’ backgrounds and clinical experience, including both patient and healthcare professional perspectives, should have affected all steps of the research process [36] and increased the likelihood of multiple stakeholders’ perspectives being maintained [37, 38]. This triangulation could enhance the credibility of the findings for both service users and health professionals [35]. Notably, the author with the patient background took part in the planning, data gathering, analysis, interpretation of data and manuscript writing phases. Nevertheless, the participants themselves did not read or comment on the results, and the findings’ credibility may thus be weakened [35].

This was an interview study, and the information provided by the patients may have been affected by recall bias or the situation in which they were interviewed [39]. Many of the interviewees were clearly aware that certain forms of answers are more socially acceptable than others in certain situations, which might also have influenced the interviews themselves [40], as might awareness of power imbalances [41]. The researchers with professional backgrounds emphasised in the interviews that they were not affiliated with the CMHCs that the participants used, and the co-researcher with lived experience as a patient participated in half of the interviews, which could reduce experienced power imbalance.

The results may be transferrable to patients with similar mental health conditions in similar settings and healthcare systems because the dynamics of communication are likely to be the same [35]. The results will be relevant to GPs, psychologists in primary mental healthcare, private healthcare doctors, emergency healthcare professionals and other professionals conducting planned, non-emergency referrals of patients to specialised mental healthcare.

## Conclusions

When asking patients how they experience the process of seeking mental healthcare, they describe various approaches to communicating their challenges according to how unrestrictedly they communicate, how receptive they are to recommendations from healthcare personnel and how active or passive they are in seeking help. The approaches to communication are dynamic and dependent on factors such as the setting, the responses of the professionals and the patient’s knowledge about mental health. Six themes describing hindrances to and facilitators of communication were also generated. To facilitate communication with patients about mental health challenges, professionals should be aware of the factors affecting communication and the interplay between those and the patient’s approach to communication. Further studies should explore how improving healthcare professionals’ communication with patients could affect decisions about the most suitable treatment for the patient from both the patients’ and professionals’ perspectives.

## Supplementary Information

Supplementary Material 1.

## Data Availability

The data that support the findings of this study are not openly available due to reasons of sensitivity and are available from the corresponding author upon reasonable request.

## Abbreviations

CMHC: Community mental health centre
GP: General practitioner
