# Supplementary material for: Conveying the need for mental healthcare – a qualitative study of how patients communicate mental health challenges
Source: BMC Health Serv Res. 2025 May 10;25:680. doi: 10.1186/s12913-025-12851-1 (PMC12065277; doi:10.1186/s12913-025-12851-1)
Supplement: Supplementary file 1 — Supplementary Material 1. [file 12913_2025_12851_MOESM1_ESM.docx]

Interview guide (will be adjusted during interviews to suit the specific situation)

Introduction:

Thank you for taking part in this study. First, I must inform you that you can withdraw your consent to participate at any time, without providing any reason, and any information you have provided will not be included in the dataset if you choose to withdraw your consent. However, if we have redacted all information that could be linked to you personally, or if the results have already been published, we will not be able to remove the information you have provided from the dataset.

In previous studies, we have investigated the healthcare system’s ability to provide timely healthcare and care tailored to individual needs. We believe that good communication between healthcare professionals and patients is crucial for receiving tailored, timely care. It is this communication that we would like to talk to you about today. This interview will provide valuable insights into how you experience communicating your need for health care when facing mental health challenges. We are interested in how you experienced communicating your need for mental health support with a general practitioner, and then with a hospital specialist at the community mental health centre (CMHC). We will also ask you whether there are factors that make it easier or more difficult to discuss your challenges with healthcare professionals at the CMHC and the GP’s office.

(General questions in the beginning of the interview)

• For how long have you been registered with your current GP?

• For how long/how many consultations did the GP provide care for the challenges that led to your referral to the CMHC? How well would you say that the GP knows you?

• When was your first consultation at the CMHC?

• Have you had any previous contact with the CMHC or any prior knowledge of the health professional providing care there?

**1. How did you experience seeking help for your mental health challenges? Please begin at the point when you decided to seek help from your GP.**

**2. How did you experience talking with your GP about the challenges or difficulties you were facing?** a. What was it about your situation that you felt was important to discuss with your GP when seeking help? How did you bring this up in the conversation? b. How did you experience the GP’s ability to listen actively to you? Did you feel the GP understood you? If so, how? How did the GP respond to what you said? c. Did you gain any new understanding after the GP consultation? Did the GP provide any new insights into your health challenges? Was the communication about your challenges clearer? If so, how? **d. Did you have any information about your health situation that you didn’t share with the GP, or that you presented differently? If so, why?**

**3. I would like to ask you the same questions regarding your experience with the first consultation at the CMHC:** a. What was important for you to share with the hospital specialist during the consultation at the CMHC? How did you experience the hospital specialist’s ability to listen actively? How did the hospital specialist understand you? b. Did you gain any new insights into your health situation after the consultation with the hospital specialist? If so, how did this affect the way you describe your challenges? c. Did you withhold any information or present any information differently to the hospital specialist? If so, why?

1. You have had to describe your challenges several times. How, if at all, has the way you describe your challenges changed over time?
2. What has helped you describe your challenges to the GP? What has hindered you in describing them to the GP? What has helped you describe your challenges to the hospital specialist? What has hindered you in describing them to the hospital specialist? (Be sure to allow enough time for the participant to answer these questions.)

A. If you could change how healthcare services treated you when you sought help, what changes would you like to see? What would you like to remain the same? What would you have liked more of?

1. Are there any other people in your informal social network or work-related network who were important in helping you describe your mental health challenges and need for healthcare? How were they important?
2. Do you have any additional information on how you experienced seeking help for mental health challenges?
